# Habituation disorders in auditory middle latency response of persistent postural-perceptual dizziness patients

**DOI:** 10.3389/fneur.2024.1366420

**Published:** 2024-03-06

**Authors:** Toshihisa Murofushi, Fumiyuki Goto, Munetaka Ushio

**Affiliations:** ^1^Department of Otolaryngology, Mizonokuchi Hospital, Teikyo University School of Medicine, Kawasaki, Japan; ^2^Department of Otolaryngology-Head and Neck Surgery, Tokai University School of Medicine, Isehara, Japan; ^3^Department of Otolaryngology, Toho University Medical Center Sakura Hospital, Sakura, Japan

**Keywords:** PPPD, vestibular migraine, habituation, potentiation, sensory gating, sensory processing disorder

## Abstract

**Objectives:**

To study habituation disorders in auditory middle latency response (AMLR) to repetitive stimuli of persistent postural-perceptual dizziness (PPPD) patients.

**Subjects:**

Twenty-eight PPPD (10 men and 18 women, mean 59.5 years of age, 26–81 years of age) were enrolled. For comparison, data of 13 definite vestibular migraine (VM) patients (3 men, 10 women, mean age 45.5), 13 definite unilateral Meniere’s disease (MD) patients (2 men, 11 women, mean age 50.6), and 8 healthy control (HC) subjects (2 men, 6 women, mean age 37.1) in the previous study were utilized.

**Methods:**

The electrodes were placed on the vertex and the spinal process of the fifth cervical vertebra. Clicks (0.1 msec, 70 dB nHL) were binaurally presented and averaged (800 times). Averaged responses were divided into 4 sets (S1 to S4) according to the temporal order. As peaks, Na, and Pa were identified, and relative Na-Pa amplitudes in S2–S4 to S1 were analyzed.

**Results:**

The mean relative amplitude of PPPD patients showed lack of habituation (potentiation) as shown in VM patients, although the extent of potentiation was weaker than VM. Comparison of relative S4 amplitudes showed significant differences among the 4 groups (*p* = 0.0013 one-way ANOVA), Multiple comparison revealed significant differences between PPPD and MD (*p* = 0.0337 Dunnet’s test).

**Conclusion:**

PPPD patients showed lack of habituation (potentiation) of Na-Pa amplitude in AMLR to repetitive stimuli. Lack of habituation (potentiation) might be associated with sensory processing disorders in PPPD.

## Introduction

Persistent postural-perceptual dizziness (PPPD) is featured by chronic dizziness and/or non-spinning vertigo, which are exacerbated in the upright position during standing or walking (1). Recently, it has been reported that functional connectivity in the central nervous system is changed in PPPD patients (2). However, its pathophysiology is still remained to be clarified.

To diagnose as having PPPD, existence of some precipitating conditions such as acute vertigo attacks is required (1). It is well known that vestibular migraine (VM) is one of major precipitating conditions of PPPD (3). As a kind of sensory processing disorders, habituation disorders in migraine have been reported (4, 5). In migraine habituation disorders have been reported to visual stimuli as well as to auditory stimuli (4, 5). Murofushi et al. have reported that VM patients show lack of habituation to repetitive stimuli in auditory middle latency responses (AMLR). AMLR is one of auditory evoked potentials which appear between 10 and 60 msec after the stimulus. The origins of AMLR were considered to exist in the thalamocortical pathway and mesencephalic reticular formation as well as the primary auditory cortex. Therefore, AMLR is one of appropriate tools for assessment of sensory processing disorders in the central nervous system. Murofushi et al. proposed that habituation test using AMLR might be useful for assessment of habituation disorders in patients with vestibular problems because such patients could show sensory processing disorders in various sensory modalities (6, 7). In this context, we hypothesized that PPPD patients might have a similar pathophysiological condition, a habituation disorder.

Herein, we preliminarily studied habituation in AMLR to repetitive stimuli in PPPD patients and compared the results with those of patients with VM, Meniere’s disease (MD) and healthy controls (HC) in the previous study (6).

## Materials and methods

### Subjects

Twenty-eight patients (10 men and 18 women, mean 59.5 years of age, 26–81 years of age) diagnosed with having PPPD according to Barany Society criteria (1) were enrolled. Also, data of patients with definite VM (*N* = 13, 3 men and 10 women, mean age 45.5, range 30–69), unilateral definite MD (*N* = 13, 2 men and 11 women, mean age 50.6, range 31–79) and HC (2 men and 6 women, mean age 37.1, range 24–50) were used for comparison (6).

### Methods

#### Recording of AMLR

Basic methods of AMLR recording were the same as the previous study (6). Briefly, AMLRs were recorded with Neuropack system (Nihon Kohden Co., Ltd., Japan). The active electrode was placed on the vertex, and the reference electrode was on the spinal process of the fifth cervical vertebra. The ground electrode was on the nasion. The subjects in the supine position were awake with the eyes closed. Clicks (0.1 msec, 70 dB nHL) were binaurally delivered through the headphone (Elga Acous Co., Ltd., Japan). Repetition rate was 5 Hz. Bandpass-filtered (20–1,000 Hz) signals were averaged. Recorded 800 responses were divided into 4 blocks. Set 1 (S1) was averaging of the first 200 responses, Set 2 (S2) the second 200 responses, Set 3 (S3) the third 200 responses, and Set 4 (S4) the fourth 200 responses. S4 amplitude/S1 amplitude <1 implies S4 amplitude is smaller than S1 amplitude. It is regarded as “habituation.” On the other hand, S4 amplitude/S1 amplitude >1 implies S4 amplitude is larger than S1 amplitude. It is regarded as “lack of habituation” in other words, “potentiation.”

As the peaks, No, Po, Na, and Pa were determined (8). As the relative amplitude of Na-Pa in S4 to S1 only showed significant difference in VM from MD and HC in the previous study (6), we focused on the relative amplitude of Na-Pa in S2–S4 to S1 in this study. The criteria for determining each peak were the same as the previous study (6).

#### Comparison of habituation in PPPD with other diseases and healthy controls

In order to compare habituation to repetitive stimuli in PPPD with other diseases and HC, data in the previous study (VM, MD, and HC) were utilized (6).

#### Questionnaires

PPPD patients also answered to 2 questionnaires, the Dizziness Handicap Inventory (DHI) (9) and the Niigata PPPD Questionnaire (NPQ), which was developed by Yagi et al. (10) as a tool for assessing the symptoms of PPPD.

#### Statistical analyses

For statistical analyses, one-way ANOVA, *t*-test, and *u*-test were used. For multiple comparison, Bonferroni’s test and Dunnet’s test were used. *p* < 0.05 was regarded as significant.

Informed consent was obtained from each subject. This study was approved by the Ethics Committee of Teikyo University (TR20-078). This study was performed in accordance with Declaration of Helsinki (1964) and its later amendments.

## Results

### Precipitating conditions of PPPD

Among the 28 PPPD patients, the precipitating conditions were vestibular neuritis (VN) in 6 patients. Idiopathic otolithic vertigo (OV) (11–14) in 9, VM in 2, idiopathic sudden hearing loss (SH) in 1 and miscellaneous or undiagnosed vertigo/dizziness in 10.

### Migraine as a comorbid disorder

Four of the 28 PPPD patients had migraine. Two were diagnosed with having VM and the other 2 had migraine independently of vertigo attacks. Twenty-four patients did not suffer from migraine.

### Relative amplitudes of Na-Pa in S2, S3, and S4 to S1. PPPD vs. other vestibular diseases and HC

**Figure 1:**
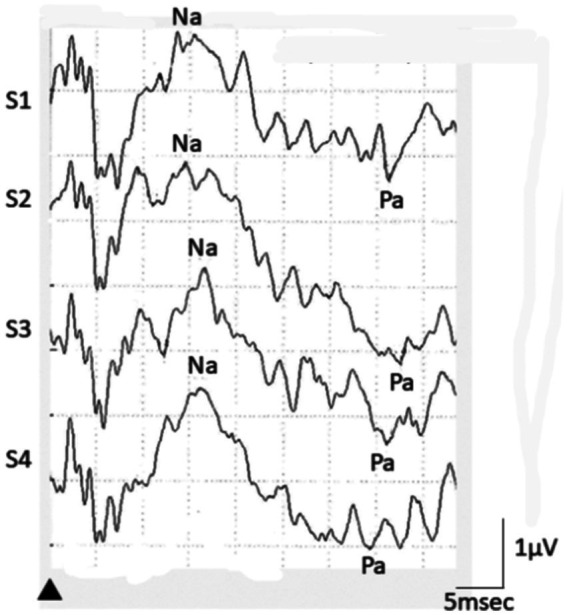
An example of AMLR recording in a PPPD patient. In this case, relative Na-Pa amplitude in S4 was 1.11 (2.47/2.22).

**Figure 2:**
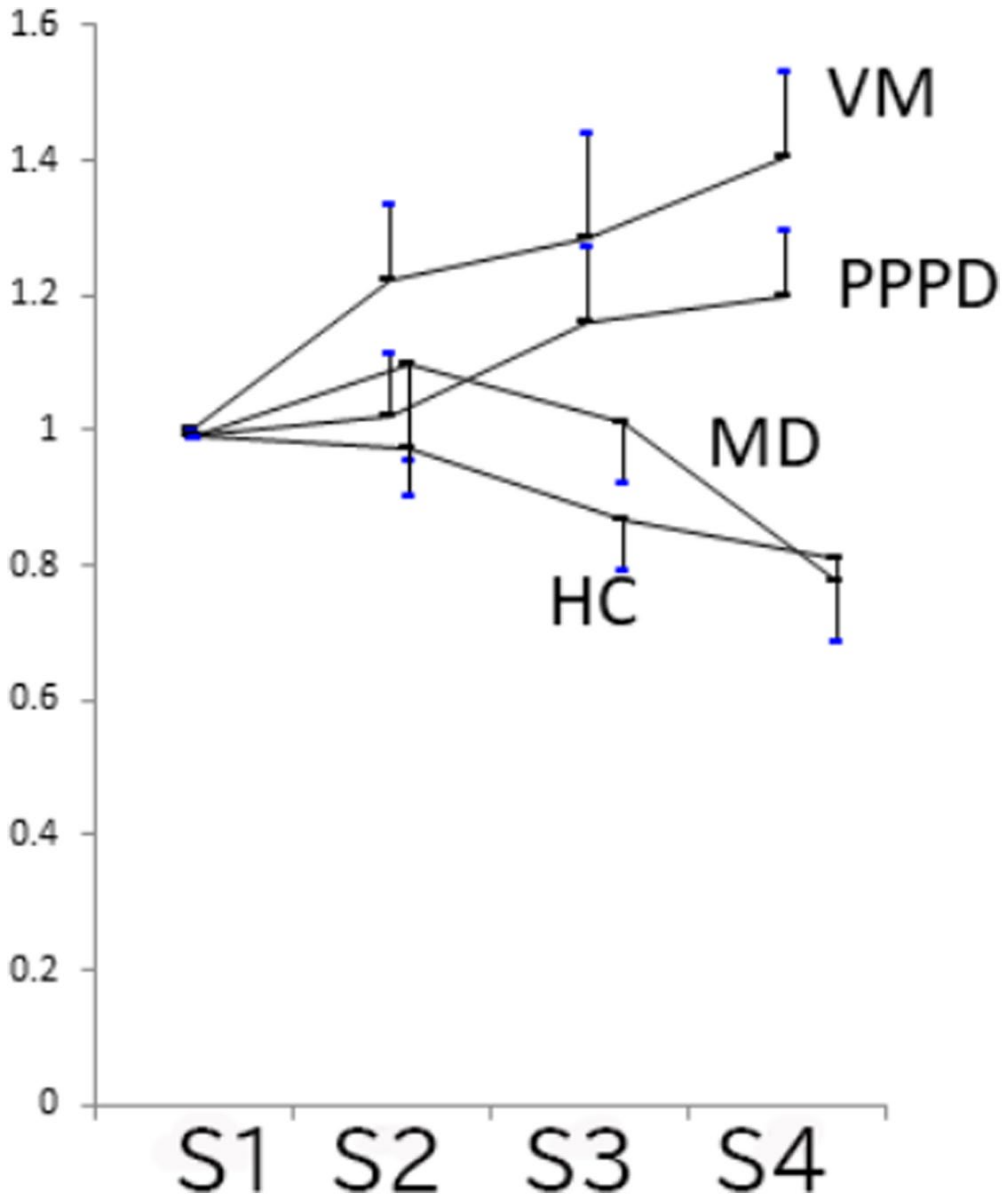
Relative Na-Pa amplitudes in S2–S4 to S1 (Mean and SE). PPPD and VM showed lack of habituation. Comparison of relative amplitudes showed significant differences among the 4 groups in S4 (*p* = 0.0013 one-way ANOVA), Multiple comparison concerning PPPD revealed significant differences between PPPD and MD (*p* = 0.0337 Dunnet’s test). Comparison between PPPD and other groups than MD (=VM and HC) did not show significant difference (*p* > 0.05 Dunnet’s test).

An example of AMLR in a PPPD patient was shown in Figure 1 Relative amplitudes of Na-Pa in S2, S3, and S4 to S1 are shown in Table 1 and Figure 2. The mean relative amplitude of PPPD patients showed lack of habituation as shown in VM patients, although the extent of potentiation was weaker than VM. Comparison of relative amplitudes showed significant differences among the 4 groups in S4 (*p* = 0.0013 one-way ANOVA), Multiple comparison revealed significant differences between PPPD and MD (*p* = 0.0337 Dunnet’s test). Also, differences between VM and MD, (*p* = 0.0091 Bonferroni’s test) and between VM and HC (*p* = 0.0422 Bonferroni’s test) were also confirmed. Comparison between PPPD and other groups than MD (=VM, HC) did not show significant difference (*p* > 0.05 Dunnet’s test).

**Table 1:** Relative amplitudes to S1 (mean ± SD).

	*N*	S2	S3	S4
PPPD	28	1.02 ± 0.09	1.16 ± 0.11	1.20 ± 0.10
VM	13	1.22 ± 0.11	1.28 ± 0.15	1.40 ± 0.31
MD	12	1.10 ± 0.13	1.01 ± 0.08	0.78 ± 0.08
HC	8	1.04 ± 0.11	0.92 ± 0.07	0.82 ± 0.11
*p*-value		NS	NS	0.0013

Because one of MD patients did not show clear AMLR, the patient was excluded.

### Comparison between PPPD patients with and without migraine

**Figure 3:**
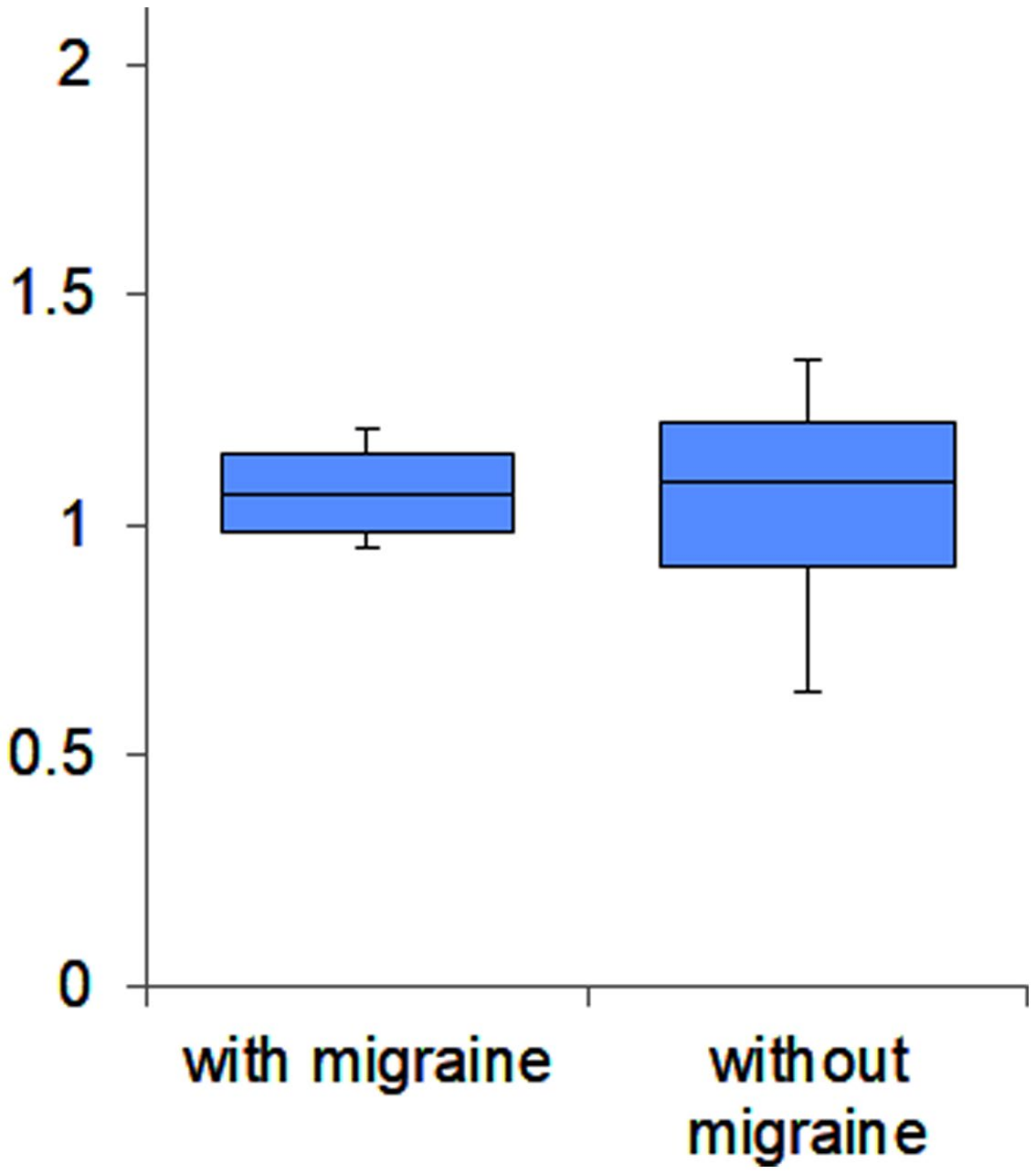
Box plots of relative Na-Pa amplitudes in S4 to S1 of PPPD patients with and without migraine. There was no significant difference (*p* > 0.05 *u*-test).

Concerning relative Na-Pa amplitude in S4 to S1, there was no significant difference between PPPD with migraine (*N* = 4) and PPPD without migraine (*N* = 24) (median 1.068 in patients with migraine and 1.090 in patients without migraine) (*p* > 0.05 *u*-test) (see Figure 3).

### Association of relative Na-Pa amplitude with questionnaire scores

There was no significant association between relative S4 amplitude and DHI score (*r* = 0.143) or between relative S4 amplitude and NPQ score (*r* = −0.028).

## Discussion

The main finding in this study was that PPPD patients showed lack of habituation (potentiation) to repetitive stimulation in AMLR. The extent of potentiation in PPPD was weaker than VM. In this study only 4 of the 28 PPPD patients suffered from migraine. The prevalence of migraine in the PPPD cohort in this study was low. It might be due to the race (Japanese) and the department (otolaryngology). Furthermore, relative S4 amplitude in PPPD patients with migraine was not different from patients without migraine. Therefore, findings in the present study can be regarded as a feature of PPPD patients.

As for origins of waves in AMLR, several areas have been proposed. Concerning wave Pa by the midline montage adopted in this study, the thalamocortical pathway and mesencephalic reticular formation as well as the primary auditory cortex are supposed as the generators (7, 15, 16). Concerning wave Na, the midbrain plays an important role (7). Therefore, lack of habituation in PPPD is considered as sensory processing disorders in the central nervous system higher than the midbrain.

Concerning lack of habituation to repetitive stimulation in AMLR of VM patients. Murofushi et al. assumed that this phenomenon might be caused by impaired “sensory gating” (6, 17). Sensory gating, a central phenomenon which is important for the processing of incoming information, causes suppression of the cortical response. This process enables the central nervous system to pay an attention selectively to important stimuli while discarding redundant stimuli (18). Habituation to repetitive stimuli and sensory gating might share common neurophysiological events. Lack of habituation and impaired sensory gating might be attributed to hypofunction of the raphe nuclei in the mesencephalic reticular formation (17).

It has been reported that SSRI (selective serotonin reuptake inhibitor), an anti-depressant, is effective for PPPD (19). This finding suggests that hypofunction of serotonergic neurons might be related to PPPD symptoms. Serotonin is synthesized in the raphe nuclei (20). Therefore, hypofunction of the raphe nuclei could cause decreased serotonin, leading to impaired sensory gating and lack of habituation to repetitive stimuli. Lack of habituation in other sensory modalities in PPPD patients should be studied as a next step.

In conclusion, PPPD patients showed lack of habituation (potentiation) of Na-Pa amplitude in AMLR to repetitive stimuli. Lack of habituation (potentiation) might be associated with sensory processing disorders in PPPD. This is a relatively small-sized preliminary study. Further large-sized study oriented to the elucidation of neurophysiological basis of sensory processing disorders in PPPD should be required.

## Data availability statement

The raw data supporting the conclusions of this article will be made available by the authors, without undue reservation.

## Ethics statement

The studies involving humans were approved by Teikyo University Ethics Committee. The studies were conducted in accordance with the local legislation and institutional requirements. The participants provided their written informed consent to participate in this study.

## Author contributions

TM: Funding acquisition, Methodology, Writing – original draft, Writing – review & editing. FG: Writing – original draft, Writing – review & editing. MU: Writing – original draft, Writing – review & editing.
